# Impact of pulmonary exposure to gold core silver nanoparticles of different size and capping agents on cardiovascular injury

**DOI:** 10.1186/s12989-016-0159-z

**Published:** 2016-08-24

**Authors:** Nathan A. Holland, Leslie C. Thompson, Achini K. Vidanapathirana, Rahkee N. Urankar, Robert M. Lust, Timothy R. Fennell, Christopher J. Wingard

**Affiliations:** 1 Department of Physiology, Brody School of Medicine at East Carolina University, Greenville, NC 27834 USA; 2 RTI International, Discovery Sciences, Research Triangle Park, NC 27709 USA

**Keywords:** Pulmonary Instillation, Myocardial Infarction, Coronary Artery, Aorta, Serum Cytokines, Nanotoxicology

## Abstract

**Background:**

The uses of engineered nanomaterials have expanded in biomedical technology and consumer manufacturing. Furthermore, pulmonary exposure to various engineered nanomaterials has, likewise, demonstrated the ability to exacerbate cardiac ischemia reperfusion (I/R) injury. However, the influence of particle size or capping agent remains unclear. In an effort to address these influences we explored response to 2 different size gold core nanosilver particles (AgNP) with two different capping agents at 2 different time points. We hypothesized that a pulmonary exposure to AgNP induces cardiovascular toxicity influenced by inflammation and vascular dysfunction resulting in expansion of cardiac I/R Injury that is sensitive to particle size and the capping agent.

**Methods:**

Male Sprague–Dawley rats were exposed to 200 μg of 20 or 110 nm polyvinylprryolidone (PVP) or citrate capped AgNP. One and 7 days following intratracheal instillation serum was analyzed for concentrations of selected cytokines; cardiac I/R injury and isolated coronary artery and aorta segment were assessed for constrictor responses and endothelial dependent relaxation and endothelial independent nitric oxide dependent relaxation.

**Results:**

AgNP instillation resulted in modest increase in selected serum cytokines with elevations in IL-2, IL-18, and IL-6. Instillation resulted in a derangement of vascular responses to constrictors serotonin or phenylephrine, as well as endothelial dependent relaxations with acetylcholine or endothelial independent relaxations by sodium nitroprusside in a capping and size dependent manner. Exposure to both 20 and 110 nm AgNP resulted in exacerbation cardiac I/R injury 1 day following IT instillation independent of capping agent with 20 nm AgNP inducing marginally greater injury. Seven days following IT instillation the expansion of I/R injury persisted but the greatest injury was associated with exposure to 110 nm PVP capped AgNP resulted in nearly a two-fold larger infarct size compared to naïve.

**Conclusions:**

Exposure to AgNP may result in vascular dysfunction, a potentially maladaptive sensitization of the immune system to respond to a secondary insult (e.g., cardiac I/R) which may drive expansion of I/R injury at 1 and 7 days following IT instillation where the extent of injury could be correlated with capping agents and AgNP size.

**Electronic supplementary material:**

The online version of this article (doi:10.1186/s12989-016-0159-z) contains supplementary material, which is available to authorized users.

## Background

The field of materials engineering has in recent decades yielded a new class of nano-sized materials. Engineered nanomaterials (ENM) are characterized by a size range of between 1 nm and 100 nm in at least one dimension, and a high surface to mass ratio [1, 2]. The diverse physiochemical properties of ENM have been utilized in a multitude of industrial, commercial and consumer applications and have raised concerns over potential human or animal toxicity. One particular class ENM of great interest include the nano-silver (AgNP) species. Nano-silver particles have innate antimicrobial properties [3] and as a result have been utilized in biomedical applications: wound dressings, silver impregnated catheters, vascular prosthetics, surgical mesh [4, 5]; and consumer applications: clothing and undergarments, air filters, laundry detergents, toiletries, and water taps [6]. The likelihood of human exposure has generated much interest in the potential toxicity of AgNP [7, 8]. Addressing concerns regarding the health impact of exposure to ENM the National Institute of Environmental Health Sciences Centers for Nanotechnology Health Implications Research (NCNHIR) Consortium was instituted to understand ENM’s biological interactions. Pulmonary responses to ENMs have been a key focus regarding investigation routes [2, 9, 10], and a large body of evidence describing AgNP and pulmonary interactions [11–15].

Despite the many investigations into the how pulmonary exposure to ENMs may impact pulmonary toxicity, there are far fewer investigations on the impact of pulmonary exposures and cardiovascular toxicity [16]. There is a strong relationship between pulmonary exposure to particulate matter and cardiovascular toxicity [1, 17–22]. It has also been demonstrated that pulmonary exposure to other forms of ENMs are capable of inducing or exacerbating cardiovascular injury [23–26]. We have recently demonstrated that pulmonary exposure to 20 nm silver-core citrate capped AgNP is capable of inducing a systemic inflammatory response, coronary artery dysfunction, and expansion of cardiac ischemia/reperfusion injury [27]. Despite these findings, the mechanisms which pulmonary exposure to AgNP may drive cardiovascular injury remain unknown. Recent studies have described toxicological responses associated with AgNP that may be strongly influenced by both particle size [14, 28] and capping agents [11, 29]. Understanding the interactions of AgNP capping as well as the influence of particle size on cardiovascular toxicity is an important, yet under investigated, step in understanding mechanisms of AgNP toxicity.

We hypothesize that intratracheal (IT) instillation of AgNP induces a systemic inflammatory response resulting in vascular dysfunction and expansion of cardiac I/R injury which is strongly dependent on particle size as well as capping agent. In order to test this hypothesis Male Sprague–Dawley (SD) rats were exposed to 200 μg of either 20 nm or 110 nm gold core AgNP capped with either citrate or polyvinylprryolidione (PVP) by intratracheal instillation. One or 7 days following AgNP instillation serum was analyzed for changes in cytokines as a marker of inflammation, subjected to cardiac ischemia/reperfusion injury and small vessel myography, evaluating aortic and coronary artery reactivity.

## Methods

### Animals

Male Sprague–Dawley (SD) rats were purchased from Charles River Laboratory (Raleigh, NC, USA) at 51–54 days of age and weighed between 201–225 g. Rats were housed two per cage under a 12 h light/dark cycle. Standard rat chow and water were provided *ad libidum*. Animals were randomly assigned to the following experimental groups for 1 day or 7 days post-instillation analysis: Naïve, Citrate Vehicle, 20 nm Au-AgNp/Citrate, 100 nm Au-AgNP/Citrate, polyvinylprryolidone (PVP) Vehicle, 20 Au-AgNP/PVP, and 110 nm Au-AgNP/PVP. Animals were allowed a 1 week acclimatization period in the East Carolina University Department of Comparative Medicine vivarium before beginning experimentation. East Carolina University’s Institutional Animal Care and Use Committee approved all animal handling and experimental procedures.

### Nanomaterial and vehicles

For the purposes of investigation both PVP and Citrate coated gold-core silver nanoparticles (AgNP) were used for instillation. The 20 nm and 110 nm AgNP were manufactured and provided to the investigators by nanoComposix (San Diego, CA) through the National Institute of Environmental Health Sciences Centers for Nanotechnology Health Implications Research (NCNHIR) Consortium funded through NIEHS. The prepared nanomaterials were independently characterized by the Nanotechnology Characterization Laboratory associated with the National Cancer Institute (Fredrick, MD) as well as independently characterized by consortium investigators [12, 30]. A summary of the nanoparticle suspension characteristics can be found in Table 1. The vehicle for PVP control groups was created by adding sterile saline to a PVP dry powder (10 and 40 Kda, Sigma-Aldrich, St. Louis, MO) to yield a 1.4 % PVP/saline solution. The vehicle control for citrate AgNP was created as a 2 mM solution of sodium citrate (Sigma-Aldrich, St. Louis, MO) dissolved in deionized water.

**Table 1:** Characterization of Au-AgNP suspensions

	Citrate Capped AgNP		PVP Capped AgNP	
	20 nm	110 nm	20 nm	110 nm
Hydrodynamic Size (nm)	24.00 ± 0.05	104.20 ± 0.12	26.00 ± 0.09	112.30 ± 0.15
Core Diameter (nm)	20.28 ± 0.23	111.3 ± 2.0	20.95 ± 0.31	114.2 ± 1.4
Zeta Potential (mV)	−48.50 ± 2.06	−43.02 ± 1.47	−37.12 ± 1.14	−25.92 ± 1.24
Silver Concentration (mg/g)	1.105 ± 0.007	0.980 ± 0.014	1.090 ± 0.001	1.101 ± 0.003
Endotoxin Concentration (EU/mL)	<0.5 ± 0	<0.5 ± 0	1.133 ± .291	<0.5 ± 0

Particle characterization data for citrate and PVP capped AgNP. Hydrodynamic size reported was determined by DLS and reported as Z-average. Core diameter was determined from TEM and Silver concentration by ICP-MS. As reported form The Nanotechnology Characterization Laboratory (NCL, Fredrick, MD) Data are as presented as a mean ± SEM of 6 separate aliquots

### AgNP suspension preparation, dosing, and intratracheal instillation (IT)

AgNP aliquots were cup-horn sonicated for 30 s at 65 % amplitude (Misonix Model 1510r-MTH, Branson Ultrasonics Corp. Danbury, CT). Silver nanoparticle and vehicle aliquots were vortexed for 30 s immediately prior to instillation. Rats were anesthetized by inhalation of a 50:50 isoflurane propylene-glycol mixture in an induction chamber. After establishment of deep anesthesia as assessed by lack of hind limb withdrawal from a toe pinch, the rat was suspended by the frontal incisors on an inclined board. The tongue was withdrawn from the oral cavity and anteriorly displaced using padded forceps and a 200 μL pipette tip containing the AgNP suspension was placed into the laryngopharynx, just superior to the glottis. Two hundred microliters of AgNP suspension containing 200 μg of AgNP or vehicle was dispensed into the glottal opening and the rat was stimulated to inhale while securing the tongue with forceps, ensuring pulmonary aspiration. The dose selected was set as a proof of concept dose agreed upon by the NCNHIR consortium members for AgNP in vivo toxicity and at this dosing could induce potential toxicological effects but not mortality or morbidity to the animal, and represents approximately 720 times the permissible exposure limit to all forms of silver at 0.01 mg/m^3^ for an 8 h work day as established by NIOSH and OSHA [31] based on rat minute ventilation rate and alveolar surface area [32]. Following instillation the rats were returned to their home cage and monitored until they resumed normal grooming behavior.

### Bronchoalveolar lavage, cell differential, and protein quantification

Sprague Dawley rats were euthanized and bronchoalveolar lavage (BAL) was performed employing a modified procedure as described by Katwa et al. [33]. Rats were anesthetized with isoflurane and euthanized by pneumothorax. The left main bronchus was ligated and a tracheotomy was performed. A 14 gauge angiocatheter was inserted into the trachea and secured with 2–0 suture. A bolus of Hanks balanced saline solution (23.1 ml/kg) was lavaged into the right lung three times successively. Recovered BAL fluid was centrifuged at 1000 × g for 10 min at 4 °C. Cell pellets were suspended in 1 mL of cold Hanks balanced saline solution. Total cell counts were determined with a Cellometer Auto ×4 (Nexcelom Biosciences, LLC, Lawrence, MA). BAL fluid volumes containing 20,000 cells were centrifuged onto glass slides using a Cytospin III (Shandon Scientific Ltd, Cheshire, UK) and stained with three-step hematology stain (Richard Allan Scientific, Kalamazoo, MI). Cell differentials were determined by microscopy counting 300 cells per slide to estimate percentage of recovered cell types. BAL supernatant was used for protein quantification using a standard Bradford protein assay. Samples were plated in duplicate using a 96-well plate. Absorbance values were read at 562 nm using a BIO-TEK Synergy HT plate reader (BIO-TEK, Winooski, VT) and data were analyzed with Gen5 software (BIO-TEK, Winooski, VT).

### Serum collection

Following anesthesia by isoflurane inhalation a cardiac puncture of the right ventricle at time of animal sacrifice was performed. Serum was separated from whole blood sample as previously described [23].

### Quantification of serum cytokines

Serum concentrations of IL-1β, IL-2, IL-5, IL-6, IL-10, IL-13, IL-18, MCP-1, G-CSF, GM-CSF, IFNγ, MCP-1, MIP-1α, RANTES, and TNFα were measured at selected time points utilizing a Milliplex MAP Cytokine/Chemokine Panel (EMD Millipore, Billerica, MA) according to the manufacturer’s instructions. Serum samples from the PVP capped AgNP and respective vehicle group were run on a Luminex 200 (Luminex, Austin, TX), while serum samples from Citrate capped AgNP and vehicle group were run on a MagPix system (Luminex, Austin, TX). All results were reported and analyzed using the Milliplex Analyst software (vVersion 5.1, EMD Millipore, Billerica, MA).

### Lung and heart protein oxidation

Heart and lavaged lung tissue was homogenized in a RIPA Buffer with 50 mM DTT and Halt™ Protease Inhibitor (Thermo Fisher Scientific, Waltham, MA). Protein concentration was determined with a Bradford Protein Assay (Bio-Rad Laboratories, Hercules, CA). Determination of protein oxidation was performed utilizing an OxyBlot™ (EMD Millipore, Billerica, MA) according to manufacturer’s instruction. Following derivatization, between 10–15 μg protein from each sample were loaded onto a Criterion Stain-Free Gel (Bio-Rad Laboratories, Hercules, CA) and electrophoresed at 200 V for 60 min. Gels were activated for 45 s with UV light using a ChemiDoc™ Touch (Bio-Rad Laboratories, Hercules, CA). Proteins were transferred utilizing a tank transfer with Towbin’s Buffer at 100 V for 60 min on to low fluorescence PVDF membranes (EMD Millipore, Billerica, MA). Membrane was imaged with a ChemiDoc™ following transfer to detect and quantify total protein. The membrane was then blocked in 1 % BSA/TBS-T for 60 min followed by incubated, at room temperature, with 1° antibody (1:150) for 60 min. The membrane was then washed with TBS-T and incubated with 2° antibody (1:300) for 1 h. The membrane was then washed with TBS-T and then incubated for 5 min in SuperSignal™ West Pico Chemiluminescent Substrate (Thermo Fisher Scientific, Waltham, MA). The membrane was imaged on a ChemiDoc™ and analyzed with Image Lab™ 5.2 (Bio-Rad Laboratories, Hercules, CA). For determination of protein oxidation analyzed density of non-derivative control lanes were subtracted from derivitized lanes, and all lanes were normalized to total protein.

### Coronary artery isolation and pharmacology

Coronary artery isolation and aorta vessel segment pharmacological response assessments were performed 1 or 7 days following IT exposure to AgNP or vehicle from animals not subject to cardiac I/R injury protocol. Isolation of the coronary artery was performed as previously described [23]. The heart and thoracic aorta was excised and placed in ice-cold physiological saline solution (PSS); [mM] 140.0 NaCl, 5.0 KCl, 1.6 CaCl_2_, 1.2 MgSO_4_, 1.2 3-[N-morpholino]-propane sulfonic acid, 5.6 d-glucose, and 0.02 EDTA (pH 7.4 @ 37 °C). Pairs of ~2 mm segments of the left anterior descending coronary artery (LAD) and aorta were excised and mounted into chambers of a 610 M multichannel myograph (DMT, Ann Arbor, MI). Coronary artery segment lumen diameter was adjusted so that resting tension was 90 % of the wall tension at 13.3 kPa while the passive force of 20 mN established for aorta segments. Tissue viability was assessed with a potassium depolarization using 109 mM K^+^PSS (Na^+^ substituted with K^+^ in an equal molar fashion). Stress generation of greater than 1 mN/mm^2^following K^+^PSS stimulation was considered adequate for vessel viability. Vessel segments were subsequently washed with PSS and endothelial integrity was assessed using a 1 μM serotonin (5-HT) or phenylephrine (PE) stimulation followed by 3 μM acetylcholine (ACh). An ACh relaxation response of > 50 % loss of the agonist stress was considered acceptable endothelial integrity. Segments were washed every 10 min with fresh PSS for a minimum of 3 times and then subject cumulative dose–response assessments [23, 34]. Paired segments were subjected to cumulative concentrations of 5-HT (10 nM−3 μM) or PE (1 nM−10 μM). The active stress (mN/mm^2^) generated in response to stimulation of paired segments was averaged at each concentration for data reporting. Upon establishing stable tension after addition of the highest agonist concentration, one of the paired segments was subject to endothelial-dependent relaxation with addition of increasing concentrations of ACh (1 nM−1 μM) and the other segment was subjected to endothelial-independent relaxation with addition of increasing concentrations of sodium nitroprusside (SNP, 1 nM−1 μM).

### Cardiac ischemia/reperfusion

One or 7 days following AgNP or vehicle intratracheal (IT) instillation rats were anesthetized with an intramuscular injection of ketamine/xylazine (90/10 mg/kg, respectively) and subject to a cardiac ischemia/reperfusion (I/R) injury [24, 27]. Anesthesia was maintained throughout the procedure with supplemental injections of ketamine/xylazine. Body temperature was maintained at 37 °C with a heating pad and TC-1000 Temperature Controller (CWE, Inc., Ardmore, PA). Once an adequate plane of anesthesia was achieved, as assessed by lack of hind limb withdrawal from a toe pinch, the rats were intubated via tracheostomy with a 16 gauge angiocatheter. Rats were then ventilated with 100 % oxygen via an Inspira Advanced Safety Ventilator (Harvard Apparatus, Holliston, MA) with setting of 3 mL tidal volume at 81 breaths per minute. Following a left parasternal thoracotomy, a temporary ligature of 6–0 vicryl suture was placed around the left anterior descending (LAD) coronary artery to induce 20 min of ischemia and removed to allow reperfusion as previously described [24].

### Quantification of infarct size

Following the ischemia reperfusion protocol the vena cava was severed and the descending thoracic aorta was cannulated with polyethelene 90 and advanced to the coronary ostia. The heart was retrograde perfused with 5 mL 0.09 % saline solution followed by 5 mL of 0.25 % TTC (Sigma-Aldrich, St. Louis, MO) to determine viable from infarcted tissue [35]. Following infusion of TTC the LAD was re-occluded and the heart infused with 1 % Evans blue dye for demarcation of remote myocardium (i.e., myocardium not subjected to the induced ischemic injury). Following Evans blue infusion, the heart was excised and sliced into approximate 1 mm thick sections distal to the occlusion. The slices were gently sandwiched between two glass slides and both sides and digitally photographed. Image J (National Institutes of Health) was used to quantify the area of the left ventricle, area at risk, and area of infarction.

### Statistical analysis

All data are presented as mean value ± SEM. A *p*-value of < 0.05 indicates statistical significance unless otherwise noted. GraphPad Prism software (Version 6, LaJolla, CA) was used for the purposes of statistical analysis and graphing. Coronary artery vascular response curves were generated using nonlinear regression analysis of the f-pair parameter best-fit values. Curves were compared using ANOVA with Tukey’s post-test for multiple comparisons. Calculated EC_50_ and Hill slope values were generated by averaging the normalized concentration-response curves (0–100 %) of individual subjects within a cohort. Results were compared using ANOVA and Tukey’s post-test for multiple comparisons. Differences between time and particle capping were performed by *t*-test. Group size was calculated based on power analysis of cardiac I/R experiments.

## Results

### Broncheolar alveolar lavage cell differentials and protein quantification

Summary of the bronchiolar alveolar lavage (BAL) data can be found in Table 2. One day following instillation of 20 nm of 110 nm citrate capped AgNP the total percentage of BAL macrophages was significantly smaller compared to the citrate control. Concurrently, a larger number of recovered epithelial cells was observed for both 20 nm of 110 nm citrate capped AgNP. There were no differences in percentage of neutrophils, eosinophils, or lymphocytes 1 day following exposure to 20 nm of 110 nm citrate capped AgNP. Additionally, there was no difference in BAL total protein concentration following instillation of citrate vehicle or citrate capped AgNP.

**Table 2:** Lung BAL cell differentials and protein

	% Macrophages	% Neutrophils	% Eosinophils	% Lymphocytes	% Epithelial Cells	Protein (mg/mL)
1 Day						
Citrate	96.0 ± 0.7	0.1 ± 0.1	0.1 ± 0.1	0.1 ± 0.1	3.8 ± 0.7	0.2 ± 0.1
20 nm AgNP	91.8 ± 1.2^*b*^	1.4 ± 1.3	0.4 ± 0.3	1.1 ± 0.3	5.3 ± 0.5	0.4 ± 0.1
110 nm AgNP	91.1 ± 0.5^*b*^	0.6 ± 0.6	0.8 ± 0.4	0.3 ± 0.1	7.0 ± 0.3^*b*^	0.4 ± 0.0
PVP	95.2 ± 0.9	0.1 ± 0.1	0.4 ± 0.3	0.8 ± 0.6	3.5 ± 0.5	0.6 ± 0.1
20 nm AgNP	92.3 ± 0.9^*b*^	0.6 ± 0.1	0.3 ± 0.2	0.3 ± 0.2	6.4 ± 1.0	0.8 ± 0.1^*b*^
110 nm AgNP	91.9 ± 0.8^*b*^	0.0 ± 0.0	0.4 ± 0.4	0.7 ± 0.2	7.0 ± 0.9	0.8 ± 0.1^*b*^
7 Day						
Citrate	88.8 ± 4.7	0.4 ± 0.2	0.1 ± 0.1	1.1 ± 0.6	9.6 ± 4.3	0.2 ± 0.0
20 nm AgNP	96.9 ± 0.9^*b*^	1.3 ± 1.0	0.2 ± 0.2	0.9 ± 0.4	0.7 ± 0.1^*b*^	0.3 ± 0.2
110 nm AgNP	96.8 ± 1.4^*b*^	0.6 ± 0.3	0.1 ± 0.1	2.0 ± 0.9	0.5 ± 0.3^*b*^	0.2 ± 0.1
PVP	95.3 ± 1.0	0.6 ± 0.3	0.1 ± 0.1	1.5 ± 0.6	2.5 ± 1.1	0.2 ± 0.0
20 nm AgNP	90.7 ± 3.9	3.6 ± 2.7	0.2 ± 0.2	3.1 ± 2.3	2.5 ± 0.9	0.2 ± 0.0
110 nm AgNP	94.2 ± 1.5	1.0 ± 0.5	0.3 ± 0.2	1.2 ± 0.6	3.3 ± 2.0	0.1 ± 0.0^*b*,*c*^

Calculated percentage of total cell BALF pellet and protein quantification. ^*b*^denotes significant (*p* < 0.05) versus vehicle, ^*c*^denotes significance from other AgNP particle size within a capping agent, calculated by one-way ANOVA with Tukey Post Hoc test Values expressed mean ± SEM, *n* = 4–8

The instillation of 20 nm or 110 nm PVP capped AgNP also resulted in a smaller percentage of BAL macrophages compared to vehicle control, with an increased number of BAL epithelial cells. Instillation of 20 nm and 110 nm PVP capped AgNP resulted in increased BAL protein concentration 1 day following exposure with no differences in percentage of neutrophils, eosinophils, or lymphocytes.

Seven days following instillation of 20 nm of 110 nm citrate capped AgNP percentage of BAL macrophages had significantly increased compared to the citrate control, and percentage of recovered epithelial cells had decreased compared to citrate control. Seven days following instillation of citrate capped AgNP resulted in no differences in percentage of neutrophils, eosinophils, lymphocytes or BAL total protein compared to vehicle control. Instillation of 20 nm and 110 nm PVP capped AgNP resulted in no changes in percentages of macrophages, neutrophils, eosinophils, or lymphocytes compared to vehicle control 7 days following instillation. Instillation of PVP capped 110 nm AgNP resulted in a lower BAL protein concentration compared to PVP vehicle PVP capped AgNP.

### Serum cytokine concentrations following exposure to Au-AGNP

**Fig. 1:**
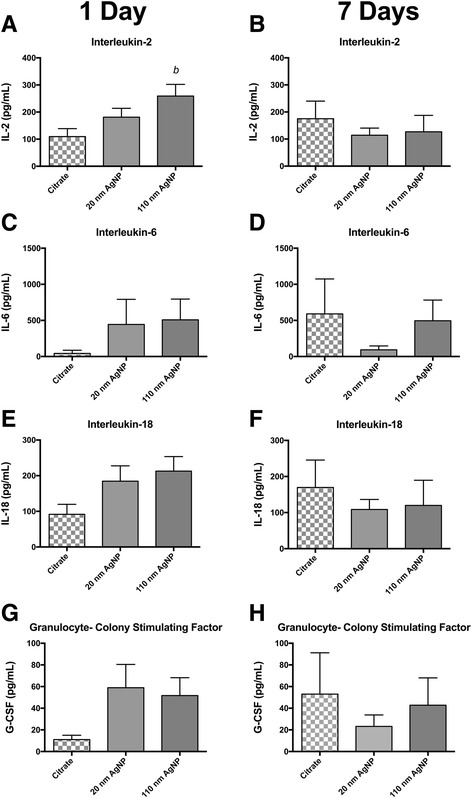
Serum Cytokine Concentrations Following IT instillation of Citrate Capped AgNP. Graphs presenenting mean serum concentration of selected cytokines 1 day (panels **a**, **c**, **e** and **g**) and 7 days (panels **b**, **d**, **f** and **h**) after IT instillation. Cytokines IL-2 (**a**) IL-6 (**c**) IL-18 (**d**) and G-CSF (**e**) were modestly elevated compared to citrate vehicle 1 day following IT instillation. At 7 days following instillation cytokine concentration was not elevated above vehicle. (*a*) denotes statistical significance from vehicle. *p* < 0.05 by one-way ANOVA, Data are reported as mean ± SEM with *n* = 4

**Fig. 2:**
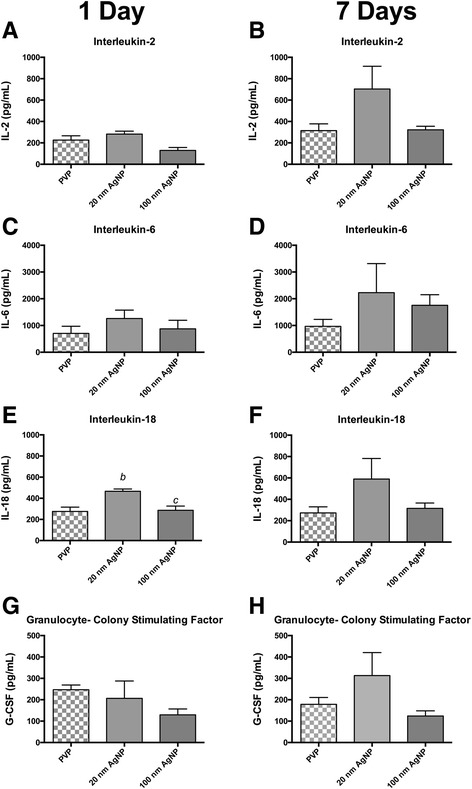
Serum Cytokine Concentrations Following IT instillation of PVP Capped AgNP. Graphs presenting mean serum concentration of selected cytokines 1 day (panels **a**, **c**, **e** and **g**) and 7 days (panels **b**, **d**, **f** and **h**) after IT instillation. Cytokines IL-2 (**a**) IL-6 (**c**) IL-18 (**d**) and G-CSF (**e**) were modestly elevated compared to citrate vehicle 1 day following IT instillation. At 7 days following instillation cytokine concentration was not elevated above vehicle. (*b*) denotes statistical significance from vehicle, (*c*) denotes significance from other AgNP particle size within a capping agent. *p* < 0.05 by one-way ANOVA, Data are reported as mean ± SEM with *n* = 4–8

Following intratracheal instillation of PVP or citrate capped 20 or 110 nm AgNP serum was collected and analyzed for concentrations of selected cytokines. The results are summarized in Figs. 1 and 2 and Additional file 1: Tables S1 and S2.

One day following IT instillation serum concentrations of IL-2 were increased by approximately 2-fold in rats instilled with 110 nm citrate capped AgNP compared to vehicle control, whereas instillation of 20 nm AgNP elicited a moderate, and non-statistically significant increase in IL-2 (Fig. 1a). Additionally, although not statistically significant, exposure to either sized citrate capped AgNPs, appears to induce equivalent yet modest increases in serum concentrations of: IL-6 (Fig. 1c), IL-18 (Fig. 1e), and G-CSF (Fig. 1g). Seven days following AgNP all elevations in serum cytokines present at 1 day were no longer evident and levels of IL-2 (Fig. 1b), IL-6 (Fig. 1d), IL-18 (Fig. 1f), and G-CSF (Fig. 1h) were comparable or below the mean values for their vehicle control. A list of all measured serum cytokines associated with the citrated capped AgNP instillation and their mean concentrations can be found in Additional file 1: Table S1.

Exposure to PVP capped AgNP also induced limited changes in serum cytokine concentrations. Pulmonary instillation of PVP capped 20 nm AgNP resulted in significant elevations in IL-18 one day following exposure, while exposure to 110 nm PVP capped AgNP did not induce increases serum concentrations of IL-18 (Fig. 2e). In contrast to the citrate capped AgNP data, 7 days post exposure PVP capped AgNP did not have a reduction in elevated cytokines at 7 days but an increase in mean values across all cytokines. Seven days post exposure to PVP capped 20 nm AgNP revealed increases in IL-2 (Fig. 2b), IL-6 (Fig. 2d), and IL-18 (Fig. 2f) compared to levels 1 day post exposure. Exposure to 110 nm AgNP did not result in values in most serum cytokines at 1 or 7 days post instillation compared to vehicle, with the exception of IL-6 that had higher value at 7 day post instillation. A list of all measured serum cytokines and their mean concentrations associated with the PVP capped AgNP instillation can be found in Additional file 1: Table S2.

### Lung and heart protein oxidation

**Fig. 3:**
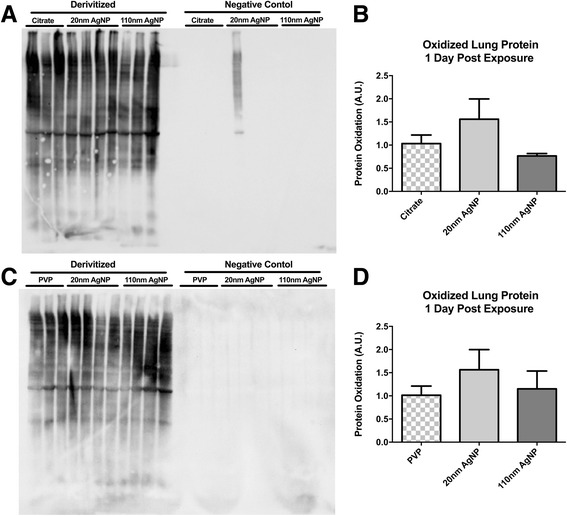
Total Lung Protein Oxidation. Representative Oxyblot™ of citrate capped AgNP exposed lung tissue (**a**). Semiquantitative densitomitry of citrate capped AgNP Oxyblot™ exposed lung tissue (**b**). Representative Oxyblot™ of PVP capped AgNP exposed lung tissue (**c**). Semiquantitative densitomitry of PVP capped AgNP Oxyblot™ exposed lung tissue (**d**). Statistical significance *p* < 0.05 by one-way ANOVA, Data are reported as mean ± SEM with *n* = 3–4

One day following instillation of 20 nm citrate capped AgNP there was a modest, but non-statistically significant increase in total lung protein oxidation compared to vehicle control as determined by OxyBlot™ assay whereas, exposure to 110 nm citrate capped AgNP resulted in a modest decrease in lung protein oxidation (Fig. 3a and b). Instillation of PVP capped 20 nm AgNP also resulted in a modest increase in lung protein oxidation compared to PVP vehicle, 110 nm PVP capped AgNP appeared to have no impact on overall lung protein oxidation (Fig. 3c and d).

**Fig. 4:**
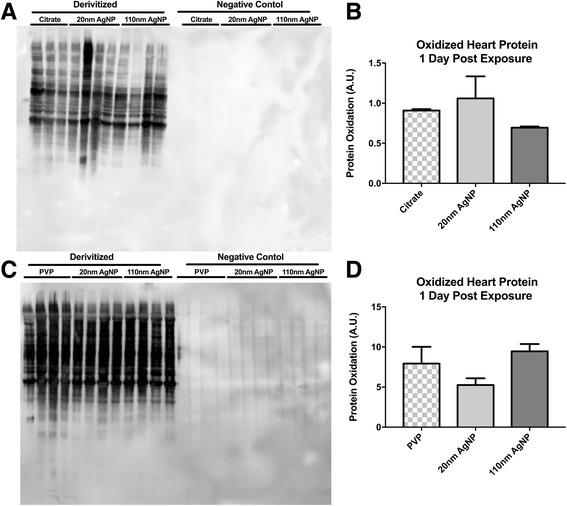
Total Heart Protein Oxidation. Representative Oxyblot™ of citrate capped AgNP exposed heart tissue (**a**). Semiquantitative densitomitry of citrate capped AgNP Oxyblot™ exposed heart tissue (**b**). Representative Oxyblot™ of PVP capped AgNP exposed heart tissue (**c**). Semiquantitative densitomitry of PVP capped AgNP Oxyblot™ exposed heart tissue (**d**). Statistical significance *p* < 0.05 by one-way ANOVA, Data are reported as mean ± SEM with *n* = 3–4

One day following instillation of 20 nm citrate capped AgNP also resulted in a minor increase in total heart protein oxidation, and exposure to 110 nm AgNP resulted in a modest reduction of heart protein oxidation compared to citrate control (Fig. 4a and b). Exposure to PVP capped 20 nm AgNP resulted in a decrease in total heart protein concentration compared to vehicle control and 110 nm PVP capped AgNP had no impact of heart protein oxidation (Fig. 4c and d).

### Au-AgNP Induced alterations in vascular responses

One day or 7 days following IT exposure to AgNP or vehicle LAD coronary artery and aorta were harvested and subjected to cumulative concentration additions of the constrictors serotonin (5-HT) or phenylephrine (PE), followed by the endothelial dependent vasodilator acetylcholine (ACh) or Nitric oxide generator sodium nitroprusside (SNP).

**Fig. 5:**
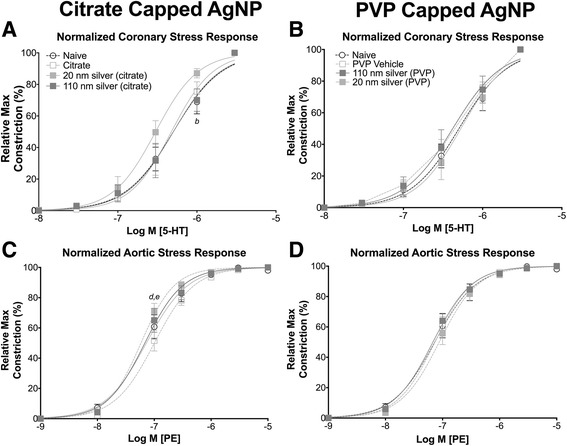
Isolated Vessel Myography Constrictor Responses 1 Day post Exposure. Graphs presenting the normalized stress responses from aorta and coronary artery segments 1 day following IT exposure including: coronary responses to citrate capped AgNP (**a**) coronary responses to PVP capped AgNP (**b**) aortic responses to citrate capped AgNP (**c**) and aortic responses to PVP capped AgNP (**d**). Statistical significance (*p* < 0.05) between: naïve and 20 nm AgNP indicated by (*b*), vehicle and 20 nm AgNP by (d), vehicle and 110 nm AgNP (*e*), calculated by two-way ANOVA with Tukey post hoc test. Lines represent the nonliner fit to the mean data. Data are reported as mean ± SEM with *n* = 3–8

One day following IT instillation, exposure to citrate capped 20 nm AgNP resulted in moderate and statistically significant, leftward shift in the concentration response curve to 5-HT for the coronary artery compared to naive, but not the vehicle control or 110 nm AgNP (Fig. 5a). Exposure to PVP capped AgNP 1 day post IT instillation did not result in a shift in the concentration response compared to vehicle or naïve controls (Fig. 7b). Exposure to 110 nm PVP capped AgNP resulted in a statistically significant reduction in calculated EC_50_ value. However instillation of all other AgNP or vehicle control, 1 day post IT exposure, resulted in no changes in maximal stress generation, calculated EC_50_, or Hill slope values in response to 5-HT in the coronary artery (Table 3).

**Table 3:** Calculated pharmacological values for coronary artery ring constrictor and relaxation response

Treatment Group	EC_50_ (nM)			Hill slope		
	5-HT	ACh	SNP	5-HT	ACh	SNP
Naive	87.7 ± 26.7	103.3 ± 28.7	20.0 ± 4.3	1.3 ± 0.1	1.8 ± 0.2	1.1 ± 0.1
1 Day Post IT Exposure						
Citrate	105.9 ± 17.1	75.6 ± 7.4	31.0 ± 5.3	1.3 ± 0.1	1.7 ± 0.1	1.2 ± 0.1
20 nm AgNP	60.2 ± 8.4	132.8 ± 56.8	22.0 ± 3.4	1.5 ± 0.1	1.5 ± 0.1	1.1 ± 0.1
110 nm AgNP	75.5 ± 16.3	147.6 ± 51.0	28.1 ± 4.8	1.4 ± 0.1	1.6 ± 0.1	1.1 ± 0.1
PVP	79.5 ± 11.5	115.7 ± 30.5	20.9 ± 3.4	1.4 ± 0.1	1.7 ± 0.2	1.2 ± 0.1
20 nm AgNP	95.1 ± 20.1	95.3 ± 3.0	18.9 ± 3.2	1.4 ± 0.1	1.5 ± 0.0	1.2 ± 0.1
110 nm AgNP	70.0 ± 10.2^*a*^	147.6 ± 51.0	23.0 ± 2.4	1.3 ± 0.1	1.8 ± 0.2	1.1 ± 0.1
7 Day Post IT Exposure						
Citrate	799.6 ± 395.9	57.4 ± 1.2	67.1 ± 33.9	1.2 ± 0.4	1.5 ± 0.0	1.0 ± 0.1
20 nm AgNP	264.2 ± 74.9	112.9 ± 29.0	55.7 ± 8.2	1.1 ± 0.2	1.6 ± 0.2	1.1 ± 0.1
110 nm AgNP	954.6 ± 80.8	98.3 ± 11.6	59.3 ± 12.5	1.1 ± 0.2	1.9 ± 0.1	1.3 ± 0.3
PVP	237.7 ± 61.1	89.2 ± 25.9	48.8 ± 3.6	1.1 ± 0.2	1.6 ± 0.3	1.2 ± 0.1
20 nm AgNP	140.8 ± 31.3	117.5 ± 26.3	41.0 ± 3.6	1.2 ± 0.2	1.5 ± 0.1	1.0 ± 0.1
110 nm AgNP	306.0 ± 70.3^*a*^	110.2 ± 39.4	50.7 ± 13.3	1.1 ± 0.2	1.6 ± 0.2	1.2 ± 0.1

The calculated mean EC_50_ and Hill slope, responses of LAD coronary artery segment to serotonin (5-HT), acetylcholine (ACh), and sodium nitroprusside (SNP) 1 day and 7 days following 200 μl instillation of Citrate or PVP vehicle or 200 μg 20 or 110 nm AgNP. Values expressed mean ± SEM. ^*a*^denotes significant versus Naïve (*p* < 0.05) calculated by one-way ANOVA with Tukey Post Hoc test, *n* = 3–8

One day post exposure to both citrate capped 20 and 110 nm AgNP resulted in a statistically significant leftward shift in concentration response curve to phenylephrine in aortic segments compared to vehicle or naïve controls (Fig. 5c). Despite the shift in concentration response curve however, there were no significant changes in aortic mean maximal stress generation, calculated EC_50_, or Hill slope values in response to PE (Table 4). Instillation of PVP capped AgNP did not alter concentration response curves to either 5-HT or PE in coronary artery or aorta segments, respectively, 1 day following exposure (Fig. 5b and d).

**Table 4:** Calculated pharmacological values for aortic ring constrictor and relaxation response

Treatment Group	EC_50_ (nM)			Hill slope		
	PE	ACh	SNP	PE	ACh	SNP
Naive	565.9 ± 129.8	127.4 ± 19.4	104.1 ± 25.3	1.7 ± 0.2	1.8 ± 0.3	2.1 ± 0.4
1 Day Post IT Exposure						
Citrate	501.9 ± 125.4	108.8 ± 47.0	74.3 ± 29.5	1.8 ± 0.2	1.4 ± 0.3	1.8 ± 0.2
20 nm AgNP	311.4 ± 58.1	97.79 ± 17.9	28.6 ± 8.0	1.7 ± 0.1	1.5 ± 0.1	1.8 ± 0.2
110 nm AgNP	558.5 ± 122.2	58.45 ± 18.1	20.9 ± 4.2	1.7 ± 0.2	1.8 ± 0.5	1.8 ± 0.3
PVP	450.2 ± 60.3	101.9 ± 20.7	44.8 ± 4.8^*a*^	1.5 ± 0.0	1.7 ± 0.1	1.7 ± 0.1
20 nm AgNP	593.0 ± 161.8	121.6 ± 31.9	40.5 ± 10.6^*a*^	1.9 ± 0.4	1.6 ± 0.1	1.8 ± 0.2
110 nm AgNP	490.8 ± 156.2	119.3 ± 20.1	20.9 ± 4.2^*a*^	1.7 ± 0.2	1.7 ± 0.4	1.9 ± 0.4
7 Day Post IT Exposure						
Citrate	497.6 ± 11.8	106.1 ± 57.9	26.4 ± 6.6^*a*^	1.8 ± 0.2	1.8 ± 0.2	1.7 ± 0.2
20 nm AgNP	256.2 ± 58.0	63.4 ± 7.4	45.1 ± 1.0^*a*^	1.6 ± 0.1	1.6 ± 0.1	1.6 ± 0.2
110 nm AgNP	540.4 ± 18.0	84.2 ± 26.3	66.3 ± 29.3	1.6 ± 0.1	1.6 ± 0.1	1.8 ± 0.1
PVP	215.0 ± 15.0	196.6 ± 82.2	40.1 ± 18.8	1.1 ± 0.1	1.5 ± 0.4	1.7 ± 0.4
20 nm AgNP	122.9 ± 14.1^*a*^	120.0 ± 13.2	66.6 ± 14.9	1.1 ± 0.1	1.1 ± 0.2	1.5 ± 0.2
110 nm AgNP	260.3 ± 50.0	66.2 ± 17.7	66.4 ± 10.6	1.1 ± 0.0	2.0 ± 0.3	1.5 ± 0.4

The calculated mean EC_50_, and Hill slope, responses of aorta segment to phenylephrine (PE), acetylcholine (ACh), and sodium nitroprusside (SNP) 1 day and 7 days post 200 μl instillation of Citrate or PVP vehicle or 200 μg 20 or 110 nm AgNP. Values expressed mean ± SEM. ^*a*^denotes significant versus Naïve (*p* < 0.05) calculated by one-way ANOVA with Tukey Post Hoc test, *n* = 3–8

**Fig. 6:**
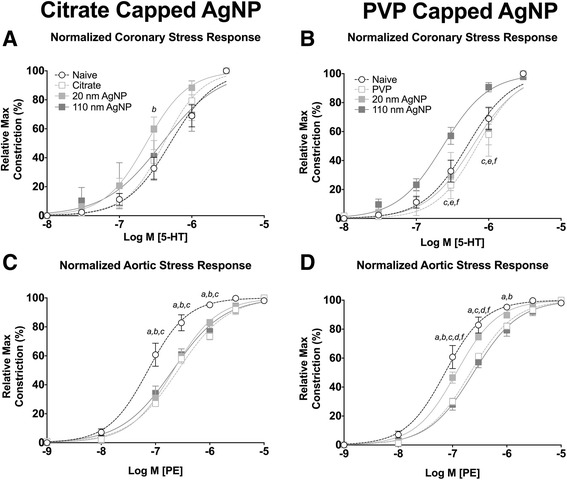
Isolated Vessel Myography Constrictor Responses 7 Days post Exposure. Graphs presenting the normalized stress responses of aorta and coronary artery segments 7 days following IT exposure including: coronary responses to citrate capped AgNP (**a**) coronary responses to PVP capped AgNP (**b**) aortic responses to citrate capped AgNP (**c**) and aortic responses to PVP capped AgNP (**d**). Statistical significance (*p* < 0.05) between: naïve and vehicle indicated by (*a*), naïve and 20 nm AgNP indicated by (*b*), naïve and 110 nm AgNP indicated by (*c*), vehicle and 20 nm AgNP, vehicle and 110 nm AgNP (*e*), 20 nm AgNP and 110 nm AgNP by (*f*), calculated by two-way ANOVA with Tukey post hoc test Lines represent the nonliner fit to the mean data. Data are reported as mean ± SEM with *n* = 3–4

Seven days following IT instillation of 20 nm citrate and 110 nm PVP capped AgNP resulted in a leftward shift in concentration response curve to 5-HT in coronary arteries (Fig. 6a and b). Interestingly, 7 days post IT instillation of citrate and PVP capped AgNP aortic segments exhibited a rightward shift in concentration response curves in response to PE (Fig. 6c and d). Exposure to AgNP or vehicle resulted in increases in mean calculated EC_50_ of aorta segments and decreases in maximal stress generation from both coronary (Table 3) and aorta segments (Table 4). No overall significant changes in Hill slope values with 5-HT or PE stimulations were observed at 1 or 7 days in either coronary or aorta segments (Tables 3 and 4).

**Fig. 7:**
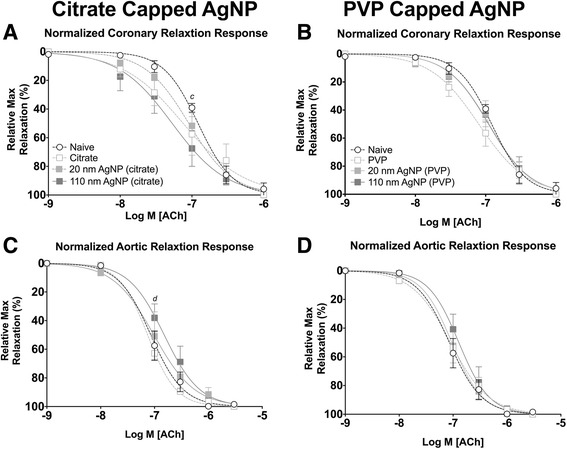
Isolated Vessel Myography ACh Responses 1 Day post Exposure. Graphs presenting the normalized stress responses of aorta and coronary artery segments 1 day following IT exposure including: coronary responses to citrate capped AgNP (**a**) coronary responses to PVP capped AgNP (**b**) aortic responses to citrate capped AgNP (**c**) and aortic responses to PVP capped AgNP (**d**). Statistical significance between naïve and 110 nm AgNP (*p* < 0.05) indicated by (*c*), vehicle and 20 nm AgNP indicated by (*d*), calculated by two-way ANOVA with Tukey post hoc test. Lines represent the nonliner fit to the mean data. Data are reported as mean ± SEM with *n* = 3–8

Endothelial dependent relaxation responses were evaluated by cumulative additions of acetylcholine (ACh). One day post IT exposure to 110 nm citrate capped AgNP resulted in a statistically significant leftward shift in the ACh concentration response curve of coronary arteries (Fig. 7a). Conversely, following IT exposure to citrate capped 110 nm AgNP there was a rightward shift in the concentration response curve to ACh in aortic rings (Fig. 7c). Intratracheal instillation of PVP AgNP did not result in significant changes to the response profiles (Fig. 7b and d) nor to the remaining stress following maximal concentration of ACh, calculated EC_50_, or Hill slope values for coronary arteries (Tables 3 and 5) or aortic rings (Tables 4 and 5) 1 day following exposure.

**Table 5:** Calculated vascular stress values for coronary arteries and aortic rings

Treatment Group	Coronary Artery			Aorta		
	Maximal Stress Generation (mN/mm^2^)	Remaining Stress (mN/mm^2^)		Maximal Stress Generation (mN/mm^2^)	Remaining Stress (mN/mm^2^)	
	5-HT	ACh	SNP	5-HT	ACh	SNP
Naive	4.0 ± 0.5	0.1 ± 0.1	0.0 ± 0.1	4.0 ± 0.5	0.1 ± 0.1	0.0 ± 0.1
1 Day Post IT Exposure						
Citrate	3.5 ± 0.4	1.2 ± 0.9	0.0 ± 0.1	3.5 ± 0.4	1.2 ± 0.9	0.0 ± 0.1
20 nm AgNP	4.3 ± 0.5	0.3 ± 0.2	0.0 ± 0.1	4.3 ± 0.5	0.3 ± 0.2	0.0 ± 0.1
110 nm AgNP	3.2 ± 0.4	0.8 ± 0.5	0.0 ± 0.1	3.2 ± 0.4	0.8 ± 0.5	0.0 ± 0.1
PVP	3.4 ± 0.3	0.4 ± 0.3	0.1 ± 0.1	3.4 ± 0.3	0.4 ± 0.3	0.1 ± 0.1
20 nm AgNP	2.7 ± 0.4	0.3 ± 0.2	0.0 ± 0.1	2.7 ± 0.4	0.3 ± 0.2	0.0 ± 0.1
110 nm AgNP	3.0 ± 0.5	0.4 ± 0.1	0.0 ± 0.1	3.0 ± 0.5	0.4 ± 0.1	0.0 ± 0.1
7 Day Post IT Exposure						
Citrate	2.4 ± 0.9	0.4 ± 0.4	−0.1 ± 0.1	2.4 ± 0.9	0.0 ± 0.1	0.6 ± 0.2
20 nm AgNP	3.6 ± 0.4	−0.1 ± 0.4	0.1 ± 0.5	3.6 ± 0.4	0.3 ± 0.1	0.4 ± 0.2
110 nm AgNP	2.8 ± 0.8	0.1 ± 0.1	−0.2 ± 0.1	2.8 ± 0.8	0.3 ± 0.3	0.0 ± 0.1
PVP	1.7 ± 0.7	0.1 ± 0.0	−0.3 ± 0.1	1.7 ± 0.7	0.5 ± 0.4	0.5 ± 0.2
20 nm AgNP	1.6 ± 0.5	0.6 ± 0.5	−0.4 ± 0.1	1.6 ± 0.5	0.6 ± 0.6	0.4 ± 0.1
110 nm AgNP	3.0 ± 0.6	0.1 ± 0.2	−0.1 ± 0.2	3.0 ± 0.6	0.5 ± 0.3	0.2 ± 0.3

The calculated mean maximum stress generated to serotonin (5-HT) or phenylephrine (PE) in coronary arteries and aortic rings respectively. Calculated remaining stress values following acetylcholine (ACh) or sodium nitroprusside (SNP) withdrawal. Values expressed mean ± SEM *n* = 3–8

**Fig. 8:**
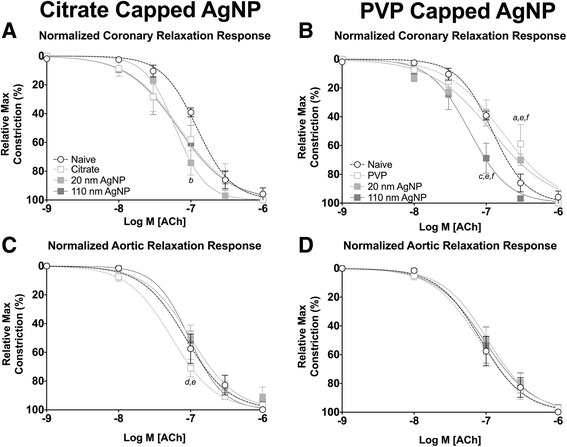
Isolated Vessel Myography ACh Responses 7 Days post Exposure. Graphs presenting the normalized stress responses of aorta and coronary artery segments 7 days following IT exposure including: coronary responses to citrate capped AgNP (**a**) coronary responses to PVP capped AgNP (**b**) aortic responses to citrate capped AgNP (**c**) and aortic responses to PVP capped AgNP (**d**). Statistical significance (*p* < 0.05) between: naïve and vehicle indicated by (*a*), naïve and 20 nm AgNP indicated by (*b*), naïve and 110 nm AgNP indicated by (*c*), vehicle and 20 nm AgNP, vehicle and 110 nm AgNP (*e*), 20 nm AgNP and 110 nm AgNP by (*f*), calculated by two-way ANOVA with Tukey post hoc test. Lines represent the nonliner fit to the mean values. Data are reported as mean ± SEM with *n* = 3–4

Seven days post IT exposure changes in response to ACh associated with Citrate capped AgNP in coronary arteries had resolved (Fig. 8a). However, exposure to 20 nm and 110 nm PVP capped AgNP resulted in a leftward shift in the concentration response curve to ACh and rightward shift to the concentration response curve to PVP vehicle following exposure in coronary arteries (Fig. 8b). No differences in calculated EC_50_, or Hill slope values were observed at 7 days following exposure when compared to naïve coronary arteries (Table 3) or aortic rings (Table 4).

**Fig. 9:**
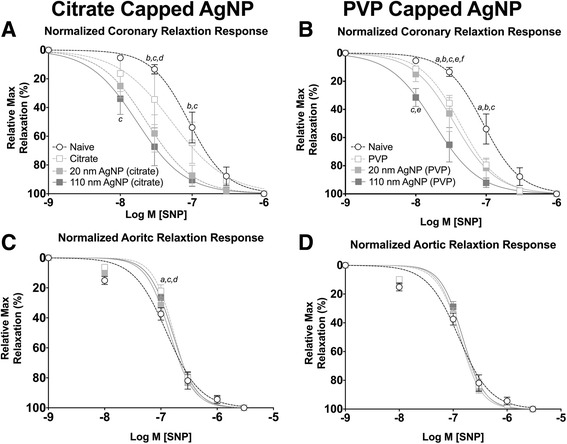
Isolated Vessel Myography SNP Responses 1 Day post Exposure. Graphs presenting the normalized stress responses of aorta and coronary artery segments 1 day following IT exposure including: coronary responses to citrate capped AgNP (**a**) coronary responses to PVP capped AgNP (**b**) aortic responses to citrate capped AgNP (**c**) and aortic responses to PVP capped AgNP (**d**). Statistical significance (*p* < 0.05) between: naïve and vehicle indicated by (*a*), naïve and 20 nm AgNP indicated by (*b*), naïve and 110 nm AgNP indicated by (*c*), vehicle and 20 nm AgNP, vehicle and 20 nm AgNP by (d), vehicle and 110 nm AgNP (*e*), 20 nm AgNP and 110 nm AgNP by (*f*), calculated by two-way ANOVA with Tukey post hoc test. Lines represent the nonliner fit to the mean values. Data are reported as mean ± SEM with *n* = 3–8

Aorta and coronary artery segments from animals exposed to AgNP or vehicle were treated with cumulative addition of SNP to assess an endothelial independent nitric oxide (NO) dependent relaxation. IT exposure to both citrate and PVP capped AgNP resulted in alterations in cumulative concentration responses to SNP in coronary arteries, 1 day following instillation (Fig. 9a and b). The 110 nm capped AgNP were capable of inducing the largest leftward shift in the concentration response curve followed by the 20 nm AgNP; however both were shifted leftward compared to vehicle or naïve in coronary arteries (Fig. 9a and b). Additionally, IT instillation of PVP vehicle was capable of inducing a small leftward shift in the concentration response to SNP in coronary arteries itself (Fig. 9b). The left shift in relaxation response profiles was accompanied by a reduction in calculated EC_50_ values for aortic rings (Table 4). However, no differences in EC_50_, Hill slope values or mean remaining stress following maximal concentration were observed in coronary artery segments 1 day following exposure (Table 3).

The sensitization effect to SNP on normalized relaxation response was only observed at 1 day post exposure post instillation of citrate capped AgNP in aortic rings (Fig. 9c and d). This is despite a significant decrease in calculated EC_50_ values of aortic rings (Table 4). However, Hill slope values, (Table 4) or remaining stress following cumulative addition of SNP (Table 5) were not different.

**Fig. 10:**
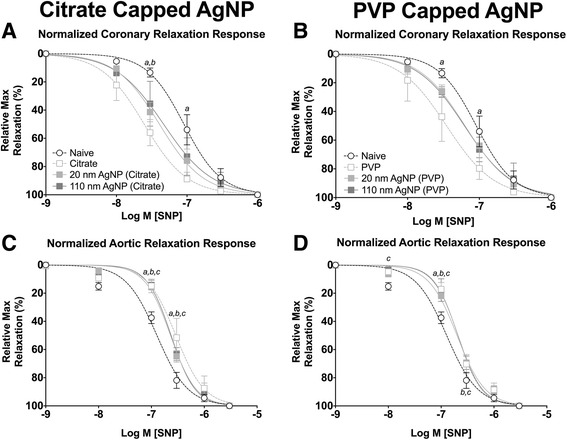
Isolated Vessel Myography SNP Responses 7 Days post Exposure. Graphs presenting the normalized stress responses of aorta and coronary artery segments 7 days following IT exposure including: coronary responses to citrate capped AgNP (**a**) coronary responses to PVP capped AgNP (**b**) aortic responses to citrate capped AgNP (**c**) and aortic responses to PVP capped AgNP (**d**). Statistical significance (*p* < 0.05) between: naïve and vehicle indicated by (*a*), naïve and 20 nm AgNP indicated by (*b*), naïve and 110 nm AgNP indicated by (*c*), calculated by two-way ANOVA with Tukey post hoc test. Lines represent the nonliner fit to the mean values. Data are reported as mean ± SEM with *n* = 3–4

Seven days following exposure to AgNP only exposure to citrate 20 nm AgNP exhibited sensitized relaxation responses to SNP in coronary arteries. However, IT instillation of both citrate and PVP vehicle were able to induce a marked leftward shift in relaxation responses to SNP in coronary arteries (Fig. 10a and b). Coronary segments from AgNP or vehicle exposed rats demonstrated a moderate decrease in calculated EC_50_ value, with no differences in Hill slope value (Table 3), or remaining stress following maximal addition of SNP (Table 5).

Aortic rings demonstrated impaired endothelial independent NO dependent relaxation 7 days post IT instillation of AgNP or vehicle compared to naïve controls (Fig. 10c and d). As observed in coronary arteries exposure to the vehicle had a marked effect on SNP stress withdrawal in aortic rings at 7 days post exposure. Post exposure aortic rings exhibited increased EC_50_ values but no differences in Hill slope value (Table 4), and a greater remaining amount of stress following maximal SNP addition as compared to naïve (Table 5).

### Au-AgNP expands cardiac i/r injury following it instillation

**Fig. 11:**
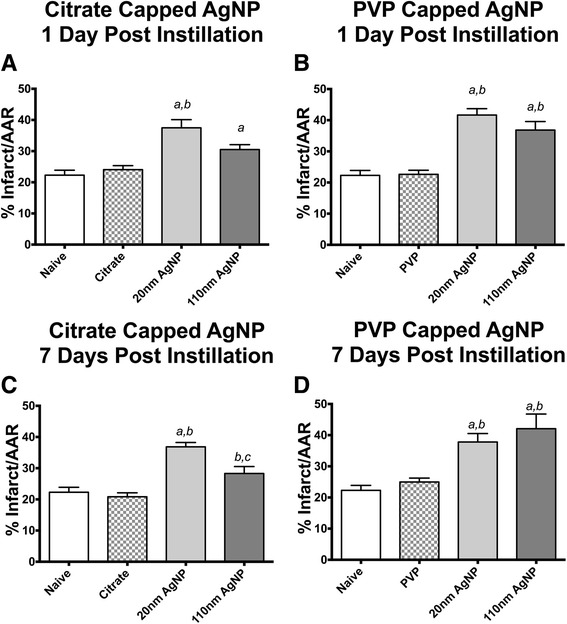
Cardiac Ischemia/Reperfusion Injury Following IT instillation of AgNP. Graphs of cardiac I/R injury in male SD rats exposed by IT instillation of 200 μl of citrate or PVP vehicle or 200 μg AgNP for 1 (panels **a** and **b**) or 7 days (panels **c** and **d**). Cardiac I/R injury was induced by occlusion of the LAD for 20 min of ischemia and 2 h of reperfusion. Ischemia-reperfusion injury was expanded 1 day following IT exposure to AgNP compared to vehicle or naïve (**a**). Expansion of I/R injury persists 7 days following IT instillation (**b**). (*a*) denotes significance, (*p* < 0.05) calculated by one-way ANOVA with Tukey post hoc test, versus naïve, (*b*) denotes significant vs vehicle, (*c*) denotes significant vs capping agent. Data are reported as mean ± SEM with *n* = 4

One or 7 days following IT exposure of AgNP or vehicle instillation rats underwent a surgically induced cardiac ischemia reperfusion (I/R) injury, in a protocol that included a 20 min ischemia period followed by 120 min of reperfusion, and were then analyzed for the extent of myocardial infarction. There was no effect of vehicle capping agents on the cardiac I/R injury 1 or 7 days post instillation (Fig. 11). One day post IT instillation both sizes of the citrate and PVP capped AgNP induced expansion of cardiac I/R injury compared to naïve control (Fig. 11a and b). Exposure to 20 nm AgNP induced expansion of cardiac I/R injury at 1 day post instillation in both capping groups compared to both vehicle and naïve. However, for the 110 nm particles only the PVP capped AgNP was significantly expanded compared to vehicle 1 day post instillation (Fig. 11b).

Seven days following IT instillation of AgNP or vehicle both PVP and citrate capped AgNP continued to elicit expansion of the cardiac I/R injury. Both citrate and PVP capped 20 nm AgNP induced greater expansion of cardiac I/R injury compared to vehicle or naïve (Fig. 11c and d). PVP capped 110 nm AgNP was also capable of expanding cardiac I/R injury compared to vehicle and naïve, but citrate capped 110 nm AgNP only expanded cardiac I/R injury compared to vehicle. Additionally, at 7 days post IT exposure to citrate capped 20 nm AgNP induced greater expansion of cardiac I/R injury that citrate capped 110 nm AgNP.

## Discussion

The rapidly expanding uses of ENMs have raised questions regarding their safety following exposure to such materials. To date, there is little consensus regarding the safety of engineered nanomaterials, the mechanisms by which they may induce a toxic response, or how these mechanisms may be influenced by particle size or modifications such as addition of capping agents. There is current literature evidence that pulmonary exposure to PM and ENMs can alter to progression of or to cardiovascular disease (CVD) and dysfunction through multiple pathways, including inflammation [15, 33, 36], autonomic dysfunction [25, 37, 38], oxidative stress [39–41] or mitochondrial dysfunction [42, 43]. We evaluated the impact of a pulmonary exposure to 2 different sized silver nanoparticles (AgNP) with different capping agents on pulmonary BAL cells and protein, serum cytokine (as a surrogate for systemic inflammation), protein oxidation in lung and heart, vascular function, and myocardial ischemia reperfusion (I/R) injury. Using a dose of 200 μg of AgNP which was selected by the NCNHIR consortium as a high exposure range and proof of concept dose in line with similar dosing used by other consortium investigators [29] capable of potential toxicological effects but not mortality or morbidity of the animals. This dose may not reflect a viable paradigm of human exposure, however this dosage is estimated to be approximately 720 times that of the limit for human exposure [31]. We hypothesized that intratracheal (IT) instillation of AgNP would result expansion of I/R injury, possibly mediated through a circulating inflammation signal and resulting in vascular dysfunction which is sensitive to particle size as well as capping agent. This hypothesis was based on the interpretation of effects of previously investigated ENMs including but not limited to: fullerenes [23], Multiwall Carbon Nanotubes (MWCNTs) [24] and most recently another species of AgNP [27].

In order to assess the inflammatory response, we evaluated BAL immune cells and total protein levels, serum levels of several cytokines and chemokines that are known to be associated with either particulate matter (PM) exposure or cardiovascular disease, as well as examining protein oxidation of lung and heart tissue. Exposure to AgNP had very little impact on increasing numbers of neutrophils, lymphocytes or eosinophils, only decreases in total percentages of macrophages were impacted at 1 day, leading to a rise in total percentage of recovered epithelial cells. However, in the absence of major changes in BAL total protein it is likely these epithelial cells reflect BAL technique rather than AgNP associated epithelial damage or increase in lung permeability. It is interesting to note that despite utilization of the same particles and a roughly equivalent dose our investigation demonstrated lower percentages of inflammatory cells at 1 and 7 days than other investigations [11, 12].

Collected serum samples revealed no observed temporal relationship between IT instillation of AgNP and circulating levels of cytokines. Although, cardiovascular disease and dysfunction are associated with increases in inflammatory cytokines such as IL-6 [44, 45] and IL-18 [46, 47] the large extent of cardiac I/R injury reported here does not correlate with the small changes in circulating cytokines. Although not directly related to cardiac I/R injury, IL-2 may mediate organ specific pro-inflammatory response through control of T-cell differentiation and control of Th2 cytokine production [48, 49]. The lack of correlation between circulating cytokines and the extent of I/R injury we report here suggests that the observed cytokine response is not likely the primary underlying mechanism for driving the expansion of cardiac injury. Increases in serum IL-2, IL-6, and IL-18 that were only observed in rats exposed to 20 nm AgNP, may indicate the influence of particle size and capping agent. Overall, we found that exposure to PVP or citrate capped 20 nm or 110 nm AgNP had minimal impact on lung and heart protein oxidation suggesting that the source of inflammation by not be related to metal induced oxidative stress, as previously observed in other studies [40, 43, 50, 51]. It is interesting that there lack of reports examining general protein oxidation in response to silver exposures in cardiac tissues. We also recognize the potential importance of lipid oxidation that may occur and the close to ties to the oxidant/antioxidant balance that was not examined in this study but likely underlie the reports of oxidative stress following exposure. Our findings may support an alternative hypothesis for the general effect of a pulmonary exposure to EM, one in which the EM may not directly induce an significant initial inflammatory response but exacerbate an inflammatory response to a secondary insult, such as been described following inoculation with lipopolysaccharide (LPS) [52] or immune sensitization in asthma models associated with both vascular dysfunction and expansion of myocardial infarction [53]. Previous studies investigating the EMN exposure and pulmonary fibrosis in rats demonstrated that exposure to nanomaterials alone were insufficient to induce fibrosis. However, inoculation with LPS following MWCNT exposure was able to induce greater fibrotic changes than LPS or MWCNT alone (25). Our data may provide evidence for the ability for AgNP to exacerbate inflammatory responses within I/R injury and ultimately expand myocardial I/R injury. Although we were unable to demonstrate a strong inflammatory response (through serum cytokine levels) following instillation of AgNP, analysis of cytokines localized to tissues of interest, such as myocardium or the vasculature could be a more enlightening target of investigation.

Overall, the limited changes in circulating cytokine concentrations post exposure to AgNP may result in priming of elements of the immune system for a secondary insult response and correlate with capping agents and particle size. Instillation of 20 nm PVP capped AgNP induced a greater rise in concentrations of select cytokines (i.e., IL-2, IL-6, IL-18) compared to citrate capped AgNP or their vehicle controls; while PVP and citrate capped AgNP 110 nm particles resulted in no significant differences from vehicle controls and in general the cardiac I/R injury was slightly greater and prolonged. These results allow us to suggest that a combination capping agent and particle size may be an important factor when evaluating the toxicity associated with exposure to AgNP [54].

The differential response to various particle sizes and capping agents on particles may be related to the content of protein coronas formed on the particles and reflect differing biological interactions [55]. The formation of unique protein coronas based on the capping material has been reported to influence the inflammatory response to AgNP by modulating the way nanomaterials interact with cells or tissues [56–58]. One study of gold nanoparticles revealed different capping agents have the ability to elicit differential effects on both in vitro and in vivo toxicity [59]. The results of this study allow us to suggest that different capping agents may have the ability to change the manner in which capped AgNP may impact toxicity [58, 60, 61] or contribute to the observed vehicle effects in our model of a cardiovascular injury response.

An additional aspect of the toxicological impact of AgNP on the cardiovascular system evaluated by this study was the duration of effect. In this study we observed changes in serum cytokines, vascular reactivity, and I/R injury that varied between particle size and capping agents as well as days post instillation. These observations may suggest a time-dependent aspect of particle size-capping interactions.

It should be noted that the impact of AgNP seems highly dependent upon the vascular bed examined, as well as being influenced by the duration of time following exposure, whereby the smaller diameter coronary vessels seem more susceptible to changes associated with exposure to AgNP at earlier time points than the aorta. This observation in itself is not unexpected and has been reported in a variety of studies following exposure to other materials [26, 62, 63]. Despite the smaller 20 nm particle seeming to have a stronger impact on inducing an inflammatory response or overall expansion of cardiac I/R injury, the larger 110 nm particle seems to impart the greatest influence of vascular reactivity. Whether or not moderate changes in vascular reactivity are driven by inflammation or may in fact drive expansion of cardiac I/R injury remain unresolved. It should also be noted that we observed vascular changes associated with IT instillation of both PVP and Citrate vehicles. This vehicle effect suggests that the capping agent may strongly influence observed endpoints. However, this study adds to a body of evidence that exposure to nanoscale particles can influence normal vascular function [23, 27, 64–66].

A fundamental question regarding the toxicity of AgNP is whether or not differences in particle number or mass dosing influences our toxicological end points. Given the different sizes of AgNP but an equivalent mass delivered in these studies it is reasonable to assume that there are higher numbers of particles in a fixed 200 μL sample of 20 nm AgNP than 110 nm AgNP. However, we demonstrated that there is no significant difference in I/R injury 24 h following IT instillation of all AgNP thus at 24 h it seems that particle number may not be strongly associated with the extent of I/R injury. Furthermore, 7 days following IT exposure a differential response is seen between the citrate and PVP capped AgNP of the same size, suggestive of influence of capping agent on the cardiac I/R injury response and not presumed differences in particle number. It is possible that the cardiac I/R endpoint may simply be insensitive to particle number induced toxicity. Results from the cytokine concentrations may indicate that surface area of AgNP along with capping agent play the more prominent roles in inducing cardiovascular toxicity. Particles enveloped by a protein corona which is influenced by capping agent have demonstrated differential granulation influencing potentially modulating cytokine release and in turn evolving immunological responses [61]. The capping agents of AgNP may further modulate the way particles interact with aforementioned biological interfaces leading to increased toxicity [59]. The influence of capping agent on cardiovascular toxicity may result from differences in surface charge, which can impact particle adsorption affinity for cellular surfaces [67] as well as cellular uptake [68]. Additionally, ionic dissolution in vivo may be a source of toxicity, although dissolution rate of silver was not measured in this study in vitro studies have examined the contribution of ion dissolution to overall toxicology [29, 30]. We have previously investigated the role of silver ion and found that within a range of ionic dissolution congruent with in vitro studies, it is unlikely that free silver ions play a large role in the cardiovascular toxicity observed in this current study [27]. The contribution of the species of silver nanomaterial to cardiovascular injury has yet to be fully evaluated, despite pure silver core species being capable of inducing expansion of cardiac ischemia-reperfusion injury [27] the magnitude of the injury was less than reported herewith gold core silver particles, raising questions of core stability contributing to in vivo ion dissolution or particle charge.

Although it does not appear citrate or PVP capped 110 nm AgNP has a strong influence on systemic inflammatory response or protein oxidation, there is a strong effect on the expansion of I/R injury. We suggest that AgNP mediated systemic inflammation is not the only mechanism contributing to expansion of I/R injury. A potential alternative mechanism of AgNP toxicity include induction of vascular dysfunction [20, 37, 69] which leads to an expansion of I/R injury following IT exposure to AgNP.

## Conclusions

The ubiquitous use of engineered nanomaterials makes understanding the potential toxicological outcomes of exposure to such materials a chief public health concern. We investigated the unique cardiovascular toxicity associated with pulmonary exposure to AgNP. In order to evaluate the cardiovascular toxicity endpoints following pulmonary instillation of AgNP we evaluated vascular responses in the aorta and coronary artery, as well as investigated the impact of AgNP on cardiac I/R injury. In an attempt to elucidate mechanisms of AgNP toxicity we also evaluated the serum inflammatory profile, which we hypothesized particle size and capping agents may drive processes leading to expansion of cardiac I/R injury. Our data reveals that IT instillation of AgNP results in expansion of cardiac I/R injury 1 day and 7 days post IT when compared to I/R injury from naïve and vehicle controls. Instillation of AgNP did not elicit a strong inflammatory response as measured by circulating serum cytokines, but I/R injury following IT instillation did result in a greater inflammatory response in PVP capped 20 nm AgNP compared to vehicle controls. The cardiovascular toxicity of AgNP seems to be complex, dependent on several factors including particle size and capping agent. The determination of the persistence of cardiac I/R injury beyond the 7 day time point may be important in understanding the potential public health impact of pulmonary exposures to AgNP.

## Additional file

Additional file 1: Table S1. Mean Serum Concentration of Selected Cytokines Post IT instillation of Citrate Capped AgNP. **Table S2**. Mean Serum Concentration of Selected Cytokines Post IT instillation of PVP Capped AgNP. (DOCX 27 kb)

## Abbreviations

5-HT: Serotonin
ACh: Acetylcholine
AgNP: Nanosilver
Au-AgNP: Gold core silver nanoparticle
BAL: Bronchoalveolar lavage
BALF: Bronchoalveolar lavage fluid
BSA: Bovine serum albumin
CVD: Cardiovascular disease
EC_50_: Half maximal effective concentration
ENM: Engineered nanomaterial
G-CSF: Granulocyte colony stimulating factor
GM-CSF: Granulocyte macrophage colony stimulating factor
I/R: Ischemia/reperfusion
IL: Interleukin
IT: Intratracheal
LAD: Left anterior descending coronary artery
LPS: Lipopolysaccharide
MCP: Monocyte chemotactic protein
MWCNT: Multiwall carbon nanotube
NCNHIR: Nanotechnology health implications research
NIOSH: National institute of occupational safety and health
NO: Nitric oxide
OSHA: Occupational safety and health administration
PE: Phenylephrine
PM: Particulate matter
PSS: Physiologic saline solution
PVDF: Polyvinylidene fluoride
PVP: Polyvinylprryolidone
SD: Sprague dawley
SNP: Sodium nitroprusside
TBS-T: Tris buffered saline w/ tween
TNF: Tumor necrosis factor
TTC: 2,3,5-Triphenyltetrazolium chloride
