# Supplementary material for: Impact of pulmonary exposure to gold core silver nanoparticles of different size and capping agents on cardiovascular injury
Source: Part Fibre Toxicol. 2016 Aug 24;13:48. doi: 10.1186/s12989-016-0159-z (PMC4997661; doi:10.1186/s12989-016-0159-z)
Supplement: Additional file 1: Table S1. — Mean Serum Concentration of Selected Cytokines Post IT instillation of Citrate Capped AgNP. Table S2. Mean Serum Concentration of Selected Cytokines Post IT instillation of PVP Capped AgNP. (DOCX 27 kb) [file 12989_2016_159_MOESM1_ESM.docx]

**Supplemental Table 1. Mean Serum Concentration of Selected Cytokines Post IT instillation of Citrate Capped AgNP**

| **Cytokine** | **Citrate (pg/mL)** | | **20 nm AgNP (pg/mL)** | | **110 nm AgNP (pg/mL)** | |
| --- | --- | --- | --- | --- | --- | --- |
|  | **1 Day** | **7 Days** | **1 Day** | **7 Days** | **1 Day** | **7 Days** |
| G-CSF | 59.0 ± 21.5 | 23.2 ± 10.5 | 51.7 ± 16.5 | 42.8 ± 25.3 | 11.1 ± 3.9 | 53.0 ± 38.2 |
| GM-CSF | ND | 10.9 ± 10.9 | 3.8 ± 3.8 | ND | ND | ND |
| IL-1β | 1.7 ± 1.4 | 11.6 ± 11.6 | 8.0 ± 4.3 | 4.1 ± 2.4 | 12.1 ± 8.7 | 11.6 ± 11.6 |
| IL-2 | 109.6 ± 29.3 | 175.1 ± 65.3 | 181.2 ± 32.7 | 114.0 ± 26.5 | 259.2 ± 42.9 *^b^* | 126.7 ± 60.7 |
| IL-5 | 243.1 ± 43.6 | 351.2 ± 79.6 | 374.3 ± 32.0 | 284.2 ± 36.6 | 385.7 ± 33.1 | 249.6 ± 75.4 |
| IL-6 | 43.3 ± 43.3 | 590.1 ± 484.2 | 444.5 ± 346.9 | 93.0 ± 53.7 | 508.6 ± 286.5 | 495.4 ± 286.0 |
| IL-10 | 23.9 ± 8.1 | 51.3 ± 31.2 | 54.5 ± 16.2 | 27.0 ± 10.5 | 74.1 ± 16.0 | 43.5 ± 26.7 |
| IL-13 | ND | 20.7 ± 20.7 | 23.7 ± 16.5 | 0.8 ± 0.8 | 17.2 ± 10.2 | 21.5 ± 13.6 |
| IL-17 | 54.6 ± 18.2 | 94.7 ± 37.9 | 98.9 ± 17.8 | 63.2 ± 17.2 | 108.3 ± 17.4 | 53.4 ± 31.7 |
| IL-18 | 91.7 ± 28.1 | 169.7 ± 75.9 | 184.7 ± 43.0 | 108.7 ± 27.7 | 212.8 ± 40.9 | 119.9 ± 69.8 |
| IFNγ | ND | 186.6 ± 186.6 | 140.7 ± 140.7 | 7.7 ± 7.7 | ND | 102.0 ± 102.0 |
| MIP-1α | 20.9 ± 3.7 | 28.2 ± 8.9 | 29.3 ± 3.4 | 19.6 ± 4.1 | 40.2 ± 4.5 | 23.1 ± 8.9 |
| TNFα | 12.6 ± 2.9 | 18.4 ± 6.2 | 18.8 ± 3.6 | 13.01 ± 2.7 | 23.7 ± 4.5 | 12.4 ± 5.5 |
| RANTES | 720.7 ± 26.8 | 662.9 ± 130.3 | 746.1 ± 92.1 | 652.8 ± 88.5 | 1192 ± 116.1 | 829.3 ± 229.8 |

ND indicates Not Detected, (*b*) denotes significant (p<0.05) versus vehicle calculated by one-way ANOVA with Tukey Post Hoc test Values expressed mean ± SEM, n = 4.

**Supplemental Table 2. Mean Serum Concentration of Selected Cytokines Post IT instillation of PVP Capped AgNP**

| **Cytokines** | **PVP (pg/mL)** | | **20 nm AgNP (pg/mL)** | | **110 nm AgNP (pg/mL)** | |
| --- | --- | --- | --- | --- | --- | --- |
|  | **1 Day** | **7 Days** | **1 Day** | **7 Days** | **1 Day** | **7 Days** |
| G-CSF | 246.7 ± 22.1 | 178.7± 32.0 | 281.8 ± 27.9 | 312.9 ± 107.9 | 129.4 ± 27.5 | 123.9 ± 24.3 |
| IL-1β | 72.4 ± 11.1 | 72.9 ± 14.9 | 48.0 ± 18.1 | 42.47 ± 15.6 | 68.6 ± 6.5 | 57.3 ± 11.0 |
| IL-2 | 226.7 ± 39.6 | 314.2 ± 63.9 | 637.7 ± 29.62 | 703.4 ± 213.0 | 356.2 ± 35.7 | 321.8 ± 33.9 |
| IL-6 | 704.1 ± 268.0 | 969.6 ± 257.7 | 1260.0 ± 316.1 | 2228 ± 1086 | 875.3 ± 321.0 | 1167.0 ± 518.4 |
| IL-18 | 275.4 ± 40.4 | 272.8 ± 56.7 | 466.2 ± 21.5 *^b^* | 589.2 ± 192.6 | 286.0 ± 39.9 *^c^* | 315.6 ± 49.7 |
| MCP-1 | 2102.9 ± 200.5 | 1995.6 ± 306.1 | 2494.0 ± 139.0 | 2606.0 ± 419.8 | 1860.0 ± 114.1 | 1816.0 ± 171.0 |
| TNFα | 55.6 ± 9.2 | 46.1 ± 7.5 | 42.6 ± 4.4 | 48.7 ± 15.9 | 22.9 ± 3.5 | 22.2 ± 3.8 |

ND indicates Not Detected, (*b*) denotes significant (p<0.05) versus vehicle, (*c*) denotes significance from other AgNP particle size within a capping agent, calculated by one-way ANOVA with Tukey Post Hoc test Values expressed mean ± SEM, n = 4-8.
